# Identification of Hub Genes and Key Pathways Associated with Anti-*VEGF* Resistant Glioblastoma Using Gene Expression Data Analysis

**DOI:** 10.3390/biom11030403

**Published:** 2021-03-09

**Authors:** Kesavan R. Arya, Ramachandran P. Bharath Chand, Chandran S. Abhinand, Achuthsankar S. Nair, Oommen V. Oommen, Perumana R. Sudhakaran

**Affiliations:** Department of Computational Biology and Bioinformatics, University of Kerala, Thiruvananthapuram, Kerala 695581, India; aaryakr@gmail.com (K.R.A.); rp.bharathchand@gmail.com (R.P.B.C.); abhinand.rohini@gmail.com (C.S.A.); sankar.achuth@gmail.com (A.S.N.); oommenvo@gmail.com (O.V.O.)

**Keywords:** vascular endothelial growth factor, glioblastoma, angiogenesis, anti-*VEGF* therapy, drug resistance, differentially expressed genes

## Abstract

Anti-*VEGF* therapy is considered to be a useful therapeutic approach in many tumors, but the low efficacy and drug resistance limit its therapeutic potential and promote tumor growth through alternative mechanisms. We reanalyzed the gene expression data of xenografts of tumors of bevacizumab-resistant glioblastoma multiforme (GBM) patients, using bioinformatics tools, to understand the molecular mechanisms of this resistance. An analysis of the gene set data from three generations of xenografts, identified as 646, 873 and 1220, differentially expressed genes (DEGs) in the first, fourth and ninth generations, respectively, of the anti-*VEGF*-resistant GBM cells. Gene Ontology (GO) and pathway enrichment analyses demonstrated that the DEGs were significantly enriched in biological processes such as angiogenesis, cell proliferation, cell migration, and apoptosis. The protein–protein interaction network and module analysis revealed 21 hub genes, which were enriched in cancer pathways, the cell cycle, the *HIF1* signaling pathway, and microRNAs in cancer. The *VEGF* pathway analysis revealed nine upregulated (*IL6*, *EGFR*, *VEGFA*, *SRC*, *CXCL8*, *PTGS2*, *IDH1*, *APP*, and *SQSTM1*) and five downregulated hub genes (*POLR2H*, *RPS3*, *UBA52*, *CCNB1*, and *UBE2C*) linked with several of the *VEGF* signaling pathway components. The survival analysis showed that three upregulated hub genes (*CXCL8*, *VEGFA*, and *IDH1*) were associated with poor survival. The results predict that these hub genes associated with the GBM resistance to bevacizumab may be potential therapeutic targets or can be biomarkers of the anti-*VEGF* resistance of GBM.

## 1. Introduction

The formation of new blood vessels from existing vessel in the postnatal life, referred to as angiogenesis, is an important process in both physiological and pathological conditions. It is a tightly regulated process involving the interplay of a number of pro- and antiangiogenic factors. Dysregulation of the balance between these factors leads to excess or inhibited angiogenesis, contributing to different pathological conditions [1,2,3]. Tumors cannot grow beyond a certain size unless they are vascularized to supply oxygen and much-needed nutrients for their growth. Angiogenic growth factors, such as Vascular Endothelial Growth Factor (*VEGF*), Fibroblast Growth Factor (*FGF*), Transforming Growth Factor (*TGF*), and Epidermal Growth Factor (*EGF*) [4], play an important role in promoting tumor angiogenesis and growth. Among them, *VEGF* is the key endothelial cell-specific mediator of angiogenesis. It induces angiogenesis by increasing the endothelial permeability, Endothelial cell (EC) proliferation, migration, survival, cell–cell contact and lumen formation. *VEGF* exerts its effects through binding with the cell surface receptors, a family of trans-membrane tyrosine kinase receptors. Its interaction with the receptor on the cell surface triggers the activation of intracellular signaling pathways and expression of a number of genes that modulate different cellular events critical to angiogenesis [5,6]. Since tumor angiogenesis is vital for growth, targeting it is considered a potential therapeutic strategy to inhibit tumor growth and development [1,7].

Bevacizumab, a humanized immunoglobulin G1 (IgG1) monoclonal antibody that selectively binds with a high affinity to human *VEGF* and neutralizes its biological activity, is one such anticancer agent [8]. The mechanisms of such anticancer effects include the direct inhibition of tumor-associated angiogenesis. However, recently, it has been noted that tumors develop a resistance to such anti-*VEGF* therapy and form capillaries, apparently through some alternative mechanism. This may be due to the activation of other pathways that have a proper connection with the downstream signaling of *VEGF*-mediated angiogenesis [9,10].

Glioblastoma is a common type of aggressive malignant brain tumor in adults characterized by histopathologic features involving necrosis and endothelial proliferation. These tumors arising from glial cells may be grouped as (i) grade I—pilocytic astrocytomas, pleomorphic xantho astrocytomas, and subependymal giant cell astrocytomas; (ii) grade II—oligodendrogliomas and astrocytomas; (iii) grade III—anaplastic oligodendrogliomas, anaplastic astrocytomas, anaplastic oligoastrocytomas, anaplastic ependymomas; and (iv) grade IV—the glioblastoma multiforme (GBM) [11]. Even though enormous therapies have been developed, the survival rate of GBM patients has not remarkably changed, and the five-year survival rate is 5.1% [12].

GBM is associated with excessive and aberrant angiogenesis, and it is characterized by rapid angiogenesis-dependent (re)growth, cell heterogeneity and extensive local tissue infiltration. With regard to treatment strategies, radiotherapy becomes ineffective, since GBM infiltrates the surrounding tissues and its complete resection is impossible. Further, the blood–brain barrier makes treatment more difficult and tumor cells found in the areas of hypoxia are resistant to radiotherapy. Bevacizumab, apart from inhibiting tumor angiogenesis by blocking *VEGF*, caused a disruption of the glioma stem cell microvascular niche and improved vascular normalization. However, glioma is quite often refractory to anti-*VEGF* therapy, and the molecular mechanisms underlying the development of drug resistance in GBM patients are not well-understood.

In recent years, high-throughput approaches have been developed to capture differentially expressed genes in various conditions, including drug resistance. Microarray-based gene expression profiling and sequence-based techniques like the RNA-seq analysis provide useful information about the differentially expressed genes, key pathways and the signature genes with respect to different conditions. Most of these datasets are now publicly available. Therefore, gene expression data-based computational approaches can be employed to characterize the genetic alterations at the genome level, which helps to identify differentially expressed genes and their possible physiological or pathological relevance. In the present study, the computational approach of expression data analysis is employed for identifying the potential genes responsible for the resistance to anti-*VEGF* therapy in glioblastoma. A number of studies have been conducted to examine the gene expression profiles of GBM patients compared with healthy controls and are made available in databases like the NCBI-GEO (National Centre for Biotechnology Information-Gene Expression Omnibus Database).

In this study, we analyzed the microarray datasets downloaded from the NCBI-GEO database of glioblastoma xenografts that developed a resistance against bevacizumab treatment and compared them with glioblastoma xenografts without bevacizumab treatment to examine whether the gene expression differed during the development of resistance to anti-*VEGF* therapy. Orthologous xenograft models are suitable to study GBM formation, progression, and investigation for potential therapeutics [13]. The data from three generations of glioblastoma xenografts were used to examine the changes in gene expression relating to tumor growth and angiogenesis in bevacizumab resistance tumors and, also, to understand whether the changes were similar, different or further changes occur as the tumor progresses, sustaining the resistance to therapy. The DEGs (differentially expressed genes) were identified from bevacizumab-treated and untreated samples. Analyses of Gene Ontology (GO) enrichment, protein–protein interaction (PPI) network, Kyoto Encyclopedia of Genes and Genomes (KEGG) pathway of DEG, and survival helped screen hub genes and their possible involvement in anti-*VEGF* resistance in GBM.

## 2. Materials and Methods

### 2.1. Microarray Data Collection and Processing

The Gene Expression Omnibus (GEO) (https://www.ncbi.nlm.nih.gov/geo/) [14] is a widely used public repository for retrieving functional genomics data comprising high-throughput gene expression data, chips, and microarrays. The gene expression profile, which is based on the GPL10558 platform (Illumina HumanHT-12 V4.0 expression bead chip, San Diego, CA, USA), deposited under accession number GSE81465 [11] was downloaded from GEO database. The data contained a total of 12 samples, including 3 independent biological replicates with control (IgG) treatments and 9 samples with drug treatments (bevacizumab), which in turn, were comprised of three independent biological replicates from three separate generations of xenografts. After getting the data, probe symbols were converted into the corresponding gene symbols using the annotation information in the platform.

### 2.2. Differential Expression Analysis and Identification of DEGs

DEGs of samples under various experimental conditions were screened using GEO2R (http://www.ncbi.nlm.nih.gov/geo/geo2r), a user-friendly web tool that allows users to compare two or more datasets in a GEO series. Probe sets without corresponding gene symbols were eliminated, and genes with more than one probe were averaged. Differential expression was analyzed separately by fixing the parameters as the default. The data with logFC (fold change) >1 and logFC (fold change) <-1 were selected as DEGs and opted for network construction. Further Bioinformatics and Evolutionary Genomics Venn diagram tool (http://bioinformatics.psb.ugent.be/webtools/Venn/) was used to draw the Venn diagrams of up- and downregulated genes to compare the DEGs.

### 2.3. GO and Pathway Functional Enrichment Analyses

GO enrichment and KEGG (Kyoto Encyclopedia of Genes and Genomes) pathway analysis of selected DEGs were conducted using the DAVID tool (Database for Annotation, Visualization and Integrated Discovery (david.ncifcrf.gov)) [15]. Gene Ontology analysis included three categories: molecular function, biological processes, and cellular component. For statistical analysis, a *t*-test (ANOVA) was done as the default setting. In the pathway functional analysis, genes were mapped to KEGG pathways, and *p*-value < 0.05 and count > 2 were set as the thresholds.

### 2.4. PPI Network Construction and Module Functional Analysis

The protein–protein interaction network was constructed using the STRING (Search Tool for the Retrieval of Interacting Genes; http://string-db.org) (version 10.0) [16] online database. PPI network of upregulated and downregulated DEGs constructed using STRING with a confidence score > 0.4 were considered statistically significant. The data were then imported into Cytoscape (version 3.7.1) [17] to visualize the protein interaction network. For identifying subgroups of genes sharing similar expression patterns across multiple conditions, module analysis was carried out [18]. From the PPI network, significant modules were extracted for analyzing interaction relationships of the DEGs using MCODE of Cytoscape [19], with default thresholds, which include a degree cut-off: 2, node score cut-off: 0.2, k-core: 2, and max depth: 100.

### 2.5. Identification of Hub Genes

Hub genes are genes with high connectivity. Hybrid centrality measure (HCM) was used to identify the hub genes. It included the centrality scores degree, closeness, betweenness, and mean degree of other interacted genes to calculate the HCM. The genes that gave the highest HCM scores were considered as hub genes, as indicated below:

Hybrid centrality measure of one gene = (degree of node + closeness + betweenness) + (∑Degree of connected nodes)/No. of connected nodes

### 2.6. Expression and Survival Analyses of Hub Genes

The web-based tool Gene Expression Profiling Interactive Analysis (GEPIA) (http://gepia.cancer-pku.cn) was employed to analyze TCGA (The Cancer Genome Atlas) and GTex (Genotype-Tissue Expression) data and provide interactive functions, such as profiling, plotting, differential expression analysis, and patient survival analysis [20]. The relationship between Hub gene expression and its significance in resistance was verified using the method of Kaplan–Meier for survival analysis. Analysis was done using GEPIA between the high- and low-expression groups, with a cut-off value of 50%. The hazard ratio with 95% confidence intervals and the log-rank *p*-value were calculated. *p* ≤ 0.05 or *p*(HR) ≤ 0.05 were considered statistically significant.

## 3. Results

### 3.1. Identification of DEGs

**Figure 1:**
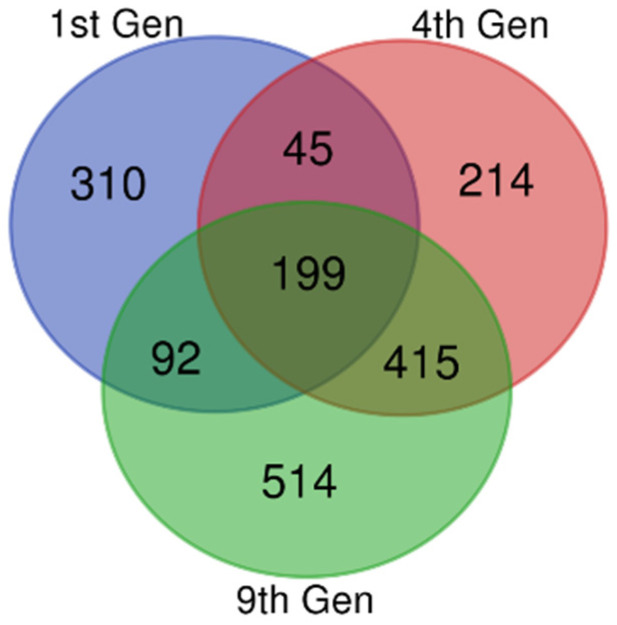
Identification of common differentially expressed genes (DEGs). Microarray data on anti-*VEGF*-resistant glioblastoma xenografts for the 1st, 4th, and 9th generations were downloaded from the Gene Expression Omnibus Database (GEO), and the DEGs were identified using GEO2R with a fold change (logFC) > 1 and logFC < −1. The Bioinformatics and Evolutionary Genomics Venn diagram tool was used to draw a Venn diagram for identifying the common genes in all the three generations. The 1st, 4th, and 9th generations were indicated as violet, red, and green, respectively. There were 199 common DEGs.

Gene expression datasets (GSE81465 from the GEO dataset for three generations) from glioblastoma xenografts, which developed resistance against bevacizumab treatment, were compared with a glioblastoma xenograft without bevacizumab controls. From the data, we extracted 646, 873, and 1220 DEGs in the first, fourth and ninth generations, respectively. There were 199 DEGs common to these samples, including 62 upregulated and 122 downregulated DEGs (Figure 1 and Table 1). The remaining 15 genes showed both up- and downregulations in resistant samples compared with the untreated controls, based on the thresholds of *p* < 0.05 and a logFC (fold change) > 1.

### 3.2. GO Function and KEGG Pathway Enrichment Analysis

**Figure 2:**
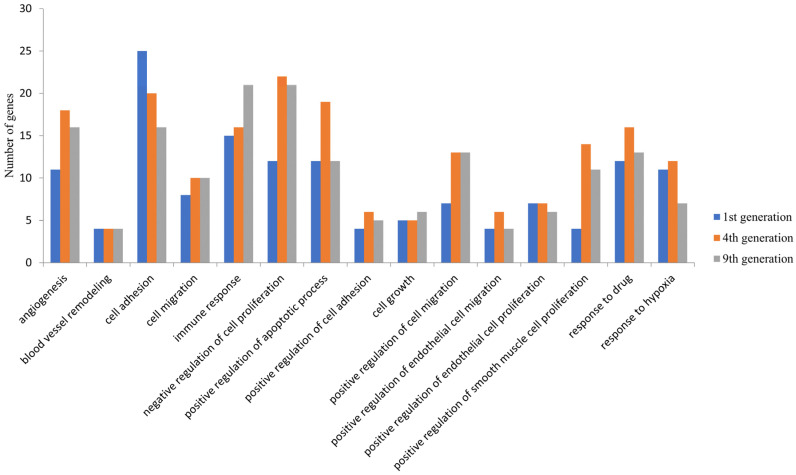
Common biological processes enrichment in upregulated DEGs in the 1st, 4th, and 9th generations. The differentially expressed genes identified were subjected to an enrichment analysis using the Database for Annotation, Visualization and Integrated Discovery (DAVID) and a set count > 2 and *p* < 0.05 as the cut-off for significant enrichment. The number of DEGs enriched for the major biological processes related to cancer and angiogenesis are presented.

The possible functions of these DEGs were examined by GO analysis using DAVID. Among the upregulated genes, the DEGs were significantly enriched in biological processes such as angiogenesis, cell proliferation and cell migration and cellular components such as the membrane and cytoplasmic components in all the three generations (Figure 2). These were enriched in molecular functions, including protein binding, receptor binding, and growth factor activity. The downregulated DEGs were significantly enriched in biological processes such as the regulation of signal transduction by the *p53* class mediator, the cell cycle and cellular components such as the cytoplasm, nucleus and nucleoplasm and molecular functions such as poly(A) RNA binding, RNA binding, and ATP binding. Apart from these common biological processes, no enrichment in other biological processes was observed in the first generation. However, the DEGS in the fourth and ninth generations were enriched in additional processes such as the negative regulation of apoptosis process, positive regulation of cell proliferation, inflammatory response, cytokine-mediated signaling pathway, and positive regulation of I-kappa B kinase/NF-kappa B signaling (Supplementary Figure S1).

**Figure 3:**
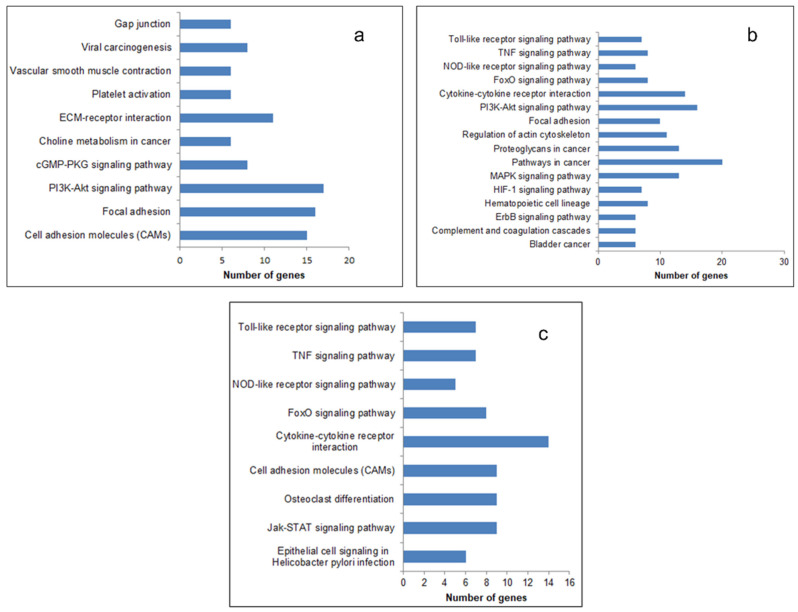
Kyoto Encyclopedia of Genes and Genomes (KEGG) pathway enrichment analysis of upregulated DEGs. The DEGs identified were subjected to a pathway enrichment analysis using DAVID and a set count > 2 and *p* < 0.05 as the cut-off for significant enrichment. The enriched pathways related to cancer and angiogenesis: (**a**) 1st generation, (**b**) 4th generation, and (**c**) 9th generation are presented.

The most significantly enriched KEGG pathways of the upregulated DEGs are presented in Figure 3. In all the three generations, the upregulated genes were enriched in the pathways that promoted cancer progression, like the Tumor Necrosis Factor *(TNF)* signaling pathway, cytokine receptor pathway, and Phosphatidylinositol 3-Kinas-Protein Kinase B (*PI3-AKT)* signaling pathway. The enriched function and pathway of downregulated genes are listed in Supplementary Figure S2. The number of pathways (27), biological processes (127), and the number of genes (368) enriched therein were the maximum in the fourth generation (Supplementary Table S1). It therefore appears that the relative resistance in terms of the number of pathways and biological processes related to cancer and angiogenesis is more in the fourth generation.

### 3.3. Classification of Differentially Expressed Genes

The DEGs identified from the fourth-generation xenografts, which showed the highest enrichment in the pathways and biological processes, could be classified by two approaches, based on their functions and roles in tumor development and progression. Together, 129 (81 up- and 48 down-) membrane-associated proteins, 66 (44 up- and 22 down-) secretory proteins, 488 (201 up- and 287 down-) intracellular protein, 43 (23 up- and 20 down-) transcription factors, and 115 (all up-) glycoproteins were identified as differentially expressed (Supplementary Table S2). Of the differentially expressed glycoproteins, 56 were glycoprotein-related enzymes, of which five were glycosyl transferases. In Table 2, we list the representative classes of the DEGs for tumor development, which involve 16 growth factors (14 up- and 2- down), 19 cytokines, including 7 interleukins and 7 proto-oncogenes.

Moreover, the GO analysis revealed that most of the angiogenic growth factors and their receptors involved in tumor progression, including *VEGF*, *EGF*, *EGFR*, and *FGF*, were differentially expressed. As the inflammatory cytokines have the potential to enhance the proliferation and invasion of GBM, we further analyzed the DEGs and identified the *IL6*, *IL1A*, and *IL1B* interleukins and 19 cytokines that were upregulated. Earlier studies revealed that Bone morphogenetic proteins (*BMPs*) and *TGFB* signaling define varying molecular and functional identities in glioblastoma [21] and also, the proto-oncogenes code for proteins, which leads to excess angiogenesis. Interestingly, we also found 4 *BMP*-related genes, 7 proto-oncogenes, and 19 receptor–ligand pairs that have a significant role in angiogenesis and also, in tumor progression.

### 3.4. PPI Network Construction, Module Analysis, and Identification of Hub Genes

**Figure 4:**
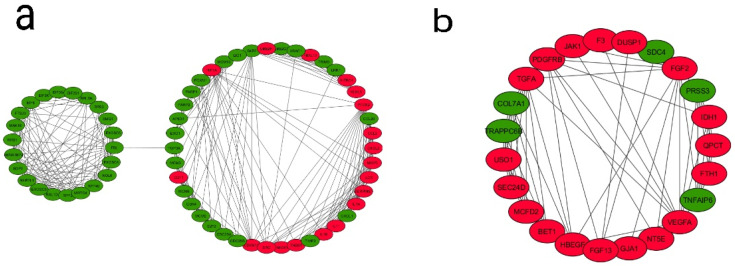
Analysis of the protein–protein interaction network of DEGs. The PPI network of DEGs were developed by the Search Tool for the Retrieval of Interacting Genes (STRING) and analyzed using Cytoscape. Modules from the PPI were extracted using the MCODE plugin in Cytoscape with default thresholds, degree cut-off: 2, node score cut-off: 0.2, k-core: 2, and max depth: 100. Seven modules with node scores > 5 were subjected to a pathway enrichment analysis—out of which, module 3 and module 4 were significant. (**a**) Module 3 with 64 nodes and 305 edges and (**b**) Module 4 with 23 nodes and 68 edges are represented. Upregulated genes are marked in red and downregulated ones in green.

The identification of protein–protein interactions on a genome-wide scale helps reveal the cellular regulation mechanisms and the function of proteins. The PPI network was constructed based on the information in the STRING database. Among the DEGs from the fourth generation, a total of 792 nodes and 5438 edges constituted the PPI network. In order to identify the most significant modules, the Cytoscape plugin MCODE with a MCODE score >5 was used. A total of seven modules were identified (Supplementary Table S3 and Supplementary Figure S3)—out of which, two modules (module 3 and 4) are enriched in pathways and genes in tumor progression and angiogenesis-related processes and, therefore, are functionally relevant (Figure 4). In module 3, out of 64 genes, 18 genes were upregulated and 46 genes downregulated. In module 4, out of 23 genes, 18 were upregulated and five downregulated. An enrichment analysis revealed that Module 3 is mainly connected with the *TNF* signaling pathway (six genes, of which four were up- and two down-), cell cycle (nine genes: one up- and eight down-), cytokine–cytokine receptor interactions (seven genes: five up- and two down-), microRNAs in cancer (seven genes: four up- and three down-), pathways in cancer (six genes: three up- and three down-), bladder cancer (five genes: three up- and two down-), *HIF1* signaling pathway (all four genes are upregulated), *NOD*-like receptor signaling pathway (three genes: two up- and one down-), and glioma (three genes: one up- and two down-). Module 4 is mainly associated with the *PI3K-Akt* signaling pathway (all five genes are upregulated), pathways in cancer (all six genes are upregulated), pancreatic cancer (all three genes are upregulated), proteoglycans in cancer (four genes: three up- and one down-), melanoma (all three genes are upregulated), *Rap1* signaling pathway (all four genes are upregulated), *Ras* signaling pathway (all four genes are upregulated), *MAPK* signaling pathway (all four genes are upregulated), and bladder cancer (both genes are upregulated) (Table 3). A similar PPI network analysis of the DEGs from the first and ninth generations was also carried out (Supplementary Tables S4 and S5). In the first generation, the module genes (out of six modules, Modules 1 and 4) were skewed towards cell adhesion molecules, Extracellular Matrix (ECM)–receptor interactions, focal adhesion, *PI3K-Akt* signaling pathway, *TNF* signaling pathway and cytokine–cytokine receptor interactions. In the ninth generation (out of 11 modules, Modules 4, 6, and 9), the number of downregulated genes associated with the modules is high, and the enriched pathways include cell cycles and metabolic pathways.

**Figure 5:**
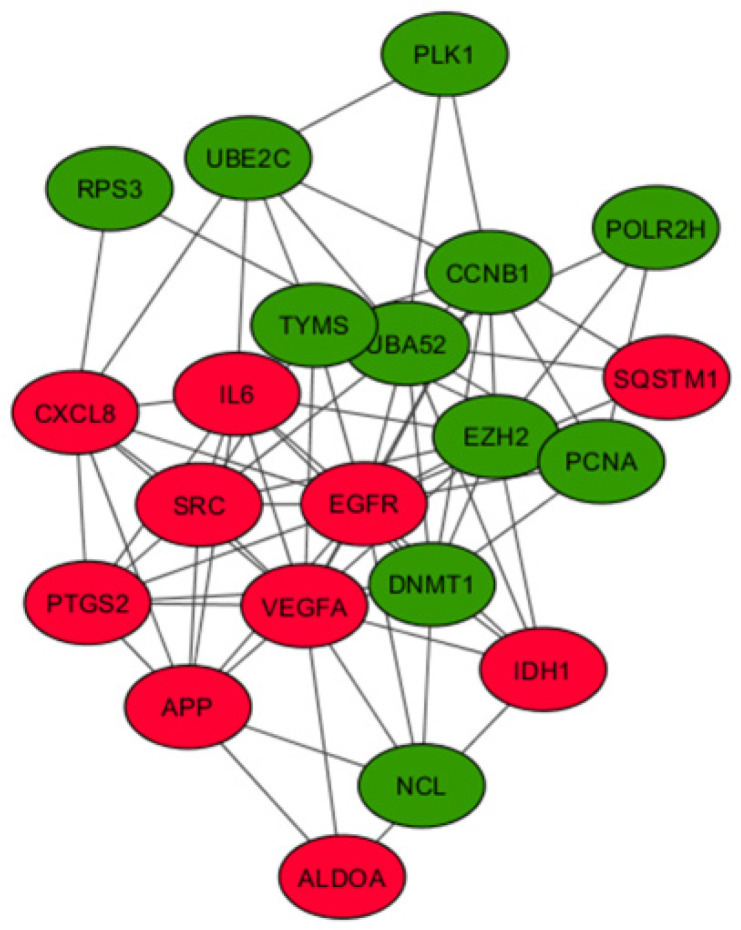
Analysis of the protein–protein interaction network for the 21 hub genes. A PPI network of 21 hub genes was constructed using STRING with a confidence score >0.4 and was considered statistically significant. Circles represent the hub genes (upregulated genes are marked in red and downregulated ones in green), and the connecting lines represent the interactions between them.

To identify the hub genes from the interaction network, a hybrid centrality measure method was employed. In the fourth generation, out of 792 nodes, 21 hub genes were finally identified with a hybrid centrality score > 12, such as *IL6*, *VEFGA*, *SRC***,** and *PTGS2* (Figure 5) and were classified into upregulated and downregulated hub genes based on the logFC values (positive logFC indicates upregulation, and negative logFC indicates downregulation) (Table 4). In the first generation, out of 553 nodes, 37 hub genes were identified (12 up- and 25 downregulated); in the ninth generation, out of 1134 nodes, 16 hub genes (6 up- and 10 downregulated) were identified (Supplementary Tables S6 and S7).

The KEGG pathway analysis of the hub genes revealed that, in the fourth generation, the hub genes were highly enriched in pathways in cancers (all five genes are upregulated), the cell cycle (all three genes are downregulated), *VEGF* signaling pathway (all three genes are upregulated), epithelial cell signaling in helicobacter pylori infection (all three genes are upregulated), microRNAs in cancer (five genes: three up- and two down-), *FoxO* signaling pathway (four genes: two up- and two down-), and in the *HIF1* signaling pathway (all three genes are upregulated) (Table 5).

However, in the first generation, the enriched pathways were skewed towards the *PI3K-Akt* signaling pathway, *HIF1* signaling pathway, Toll-like receptor signaling, *TNF* signaling pathway, *NOD*-like receptor signaling pathway, pathways in cancer, transcriptional misregulation in cancer and microRNAs in cancer, but most of the hub genes associated with these pathways were downregulated (Supplementary Table S8). However, in the ninth generation, the hub genes are only enriched in three pathways, viz., the *FoxO* signaling pathway, *HIF1* signaling pathway and the cell cycle (Supplementary Table S9).

### 3.5. VEGF Pathway Association

The interaction between hub genes and the genes in the *VEGF* pathway was analyzed for identifying altered pathways or genes in *VEGF* signaling (Supplementary Table S10). Among the 21 hub genes identified in the fourth generation, five were already reported in the *VEGF* signaling pathway. Thirteen hub genes showed interactions with 15 genes in the *PI3K-AKT-MTOR* module and with 7 genes in the *ERK* module, 11 hub genes showed interactions with 3 genes in the *NFKB* module and with 3 genes in the *P38 MAPK* module, 6 hub genes showed interactions with 10 genes in the *RAC* module, 7 hub genes showed interactions with 6 genes in the *PLC-PKC* module, and 8 hub genes showed interactions with 3 genes in the *STAT* module.

### 3.6. Survival Analysis of Hub Genes

**Figure 6:**
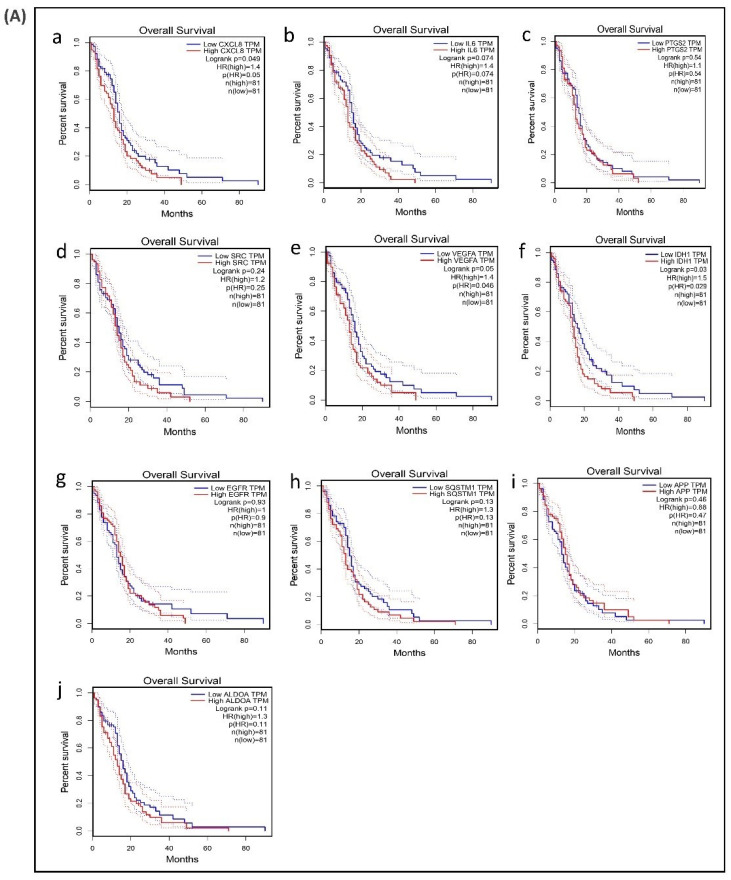

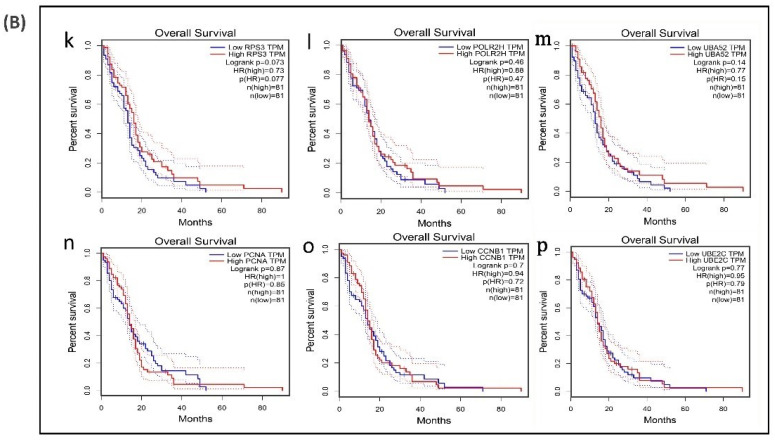
Survival analysis of differentially expressed hub genes in patients with GBM. The relationship between the expression of hub genes and survival as analyzed by plotting high and low expression levels of up- and downregulated hub genes in patients with GBM. The survival curves were plotted using the Gene Expression Profiling Interactive Analysis (GEPIA). The specific DEG expression levels were dichotomized by a median value. The results are presented visually by Kaplan–Meier survival plots. *p*-values were calculated using log-rank statistics. GBM, glioblastoma; HR, hazard ratio; and TPM, transcripts per million. (**A**) Survival plot of upregulated hub genes: (**a**) *CXCL8,* (**b**) *IL6,* (**c**) *PTGS2,* (**d**) *SRC,* (**e**) *VEGFA,* (**f**) *IDH1,* (**g**) *EGFR,* (**h**) *SQSTM1,* (**i**) *APP,* and (**j**) *ALDOA*. (**B**) Survival plot of downregulated hub genes: (**k**) *POLR2H,* (**l**) *RPS3,* (**m**) *UBA52,* (**n**) *PCNA,* (**o**) *CCNB1,* and (**p**) *UBE2C*.

A survival analysis of the 21 hub genes was performed to examine the association between the expression of each gene and the overall survival time of patients with GBM (Table 6). These results revealed that three hub genes with higher expression levels (*VEGFA*, *CXCL8*, and *IDH1*) were associated with a significantly shorter overall survival time among patients with GBM (log-rank *p* ≤ 0.05 and *p*(HR or hazard ratio) ≤ 0.05) (Figure 6A,B), suggesting that these hub genes are associated with the pathophysiology of bevacizumab-resistant GBM. However, no significant correlation was found between the expression of the other seven upregulated hub genes and six downregulated hub genes (log-rank *p* > 0.05 and *p*(HR) > 0.05). Though the low expression levels of the hub genes *EZH2*, *TYMS*, *PLK1*, *NCL*, and *DNMT1* were associated with a better overall survival, the survival analysis of the expression of these hub genes did not show a statistically significant (*p* > 0.05 and *p*(HR > 0.05) association (Supplementary Figure S4).

## 4. Discussion

Tumor angiogenesis, being a critical process for tumor growth and progression targeting, is recognized as an important therapeutic strategy. Though anti-*VEGF* therapy using inhibitors of *VEGF* action has been reported to arrest tumor growth in several types of cancers, in certain cases, resistance to antiangiogenic treatment, particularly against anti-*VEGF* therapy, has been reported. Glioblastoma is one such tumor that shows resistance to anti-*VEGF* therapy. In our effort to understand the molecular basis of resistance to anti-*VEGF* therapy in glioblastoma, we analyzed the gene expression data in xenografts from anti-*VEGF*-resistant GBM, using bioinformatics tools, and the results suggested that the cells adapt to such conditions by changing gene expression and restoring angiogenesis. This is evidenced by the following observations: (a) The analysis of the microarray data from fourth generation xenografts of anti-*VEGF*-resistant GBM patients showed the upregulation of 359 genes and downregulation of 514 genes, indicating differences in gene expression during the development of anti-*VEGF* resistance. (b) The GO function and pathway enrichment analysis of DEG showed significant enrichment in the biological processes such as cell proliferation, cell migration and angiogenesis, indicating the ability to acquire angiogenic phenotypes. A further analysis of the DEGs showed enrichment in the molecular functions such as receptor binding and growth factor activity and the signaling pathways such as *TNF* signaling pathway, *PI3-AKT* pathway, and cytokine receptor pathway, particularly in upregulated DEGs. (c) The PPI network analysis showed enrichment in the key angiogenic pathways, such as the *HIF1* pathway, *PI3-AKT* pathway and cell cycle pathway, critical in angiogenesis and cancer development. (d) Among the DEGs, several hub genes, including *IL6*, *VEGFA*, and *SRC* were identified. The survival analysis showed that the high expression of three hub genes were associated with a shorter overall survival time of GB patients.

Identification of DEGs in the resistance condition provides valuable information about the mechanism of resistance. In the present study, the gene expression profile dataset, (anti angiogenic therapy resistance condition) GSE81465 from GEO was analyzed to obtain DEGs. The GO biological process analysis revealed that several of the upregulated DEGs were functionally enriched in the process of cell proliferation, migration, cell adhesion and angiogenesis, confirming that the cells were resistant to the bevacizumab therapy and equipped to develop new vessels needed for tumor growth. Further, the downregulated DEGs were enriched in tumor suppressive pathways that regulate cell cycle and signal transduction by *p53*, indicating that the tumor growth was not arrested by anti-angiogenic therapy. Previous studies reported that anti *VEGF* therapy only seizes *VEGF* and it does not block other molecules involved in angiogenesis pathway that leads to the cell proliferation, migration and survival [22]. It was observed that 18 angiogenesis related genes were upregulated, among them *VEGF* and *TGFA* are growth factors and *NRP2* is a receptor for *VEGFA*. Several of these DEGs encode proteins that are reported to affect growth and characteristics of the GBM. For instance, *EPAS1/HIF2A* is a hypoxia responsive transcription factor, the over expression of it in glioblastoma enhances the tumor aggressiveness [23]. Many aggressive aspects of GBM such as cell proliferation and poor prognosis are highly correlated with the expression of *PTGS2* [24,25,26] and it is overexpressed in radiation resistance glioma [27]. High expression of *TNFRSF12A* has been reported in GBM [28] and is also involved in glioma cell migration, invasion, and resistance to chemotherapeutic agents. Temozolomide- resistant GBM shows high expression of *TNFRSF12A* and greater migratory capacity [29]. *CXCL8/IL8* is a multifunctional cytokine which enhances the vascular permeability in GBM [30,31]; high expression of both *VEGFA* and *CXCL8* can reduce the overall survival rate of GBM patients [32]. *ADGRG1/GPR56* is a *GPCR* involved in adhesion signaling and *HIF1A* is a transcription factor, which has a critical role in GBM survival, resistance and invasion [33]. Recent studies showed that *SRPX2* promotes epithelial to mesenchymal transition in GBM, and it’s over expression induced TMZ resistance in GBM [34]. In GBM, *EREG* enhances the phosphorylation of *EGFR*, thus activates *EGFR* signaling and directs cancer cell proliferation [35]. *CLIC4*, which is a key element in the apoptotic response to oxidative stress, is highly expressed in GBM [36].

Further analysis of the DEGs identified 15 upregulated genes associated with growth factor activity, including *CSF3*, *IL6*, *OSGIN2*, *FGF13*, *IL11*, *TIMP1*, *LIF*, *BDNF*, *EREG*, *CLCF1*, *VEGFA*, *TGFA*, *HBEGF*, *NRG1* and *FGF2*. Among these, *CSF3*, *IL6*, *IL11*, *LIF* and *CLCF1* are cytokines. In that, *FGF2* and *FGF13* showed high expression in GBM samples. Previous studies indicated that *FGF13* regulates GBM cell invasion and bevacizumab-induced glioma invasion [37,38,39]. Seven proto-oncogenes (*FYN*, *MLLT11*, *PDGFRB*, *BCL6*, *SRC*, *CBLB* and *CRKL*) were also upregulated in GBM, agreeing with the previous studies [40,41,42,43,44,45,46,47].

Pathway enrichment analysis showed that the upregulated DEGs were enriched in the cancer related pathways, suggesting that the genes involved in these pathways might be responsible for the formation of resistance to anti *VEGF* therapy in GBM. Sixteen genes in *PI3K-Akt* pathway were upregulated to suggest that the pathway was activated in GBM, possibly causing the suppression of cell death and increasing cell survival [48,49,50,51,52,53]. Twenty genes in cancer pathways were upregulated; these genes are known to have important role in biological processes such as angiogenesis, cell invasion, cell proliferation, apoptosis and mobility [52,53]. In the context of the reported role of cytokines in the glioma formation, data showing upregulation of 14 genes in the cytokine receptor interaction might be important in the induction of resistance. Upregulation of 13 genes encoding components of *MAPK* network, which is severely altered in GBM [54], has also been observed. Ten genes involved in focal adhesion were also significantly upregulated during the drug resistance condition.

Further analysis of the DEGs in terms of the nature and distribution of the proteins encoded by these genes revealed that several of them belonged to classes of glycoproteins, secretory proteins, membrane associated proteins and intracellular proteins. Alterations in glycoproteins, particularly changes in their nature and distribution have been known to play a key role in tumor development as well as resistance to drug treatment [55]. In the current study we observed that 115 glycoproteins were upregulated, most of which are present on the cell surface that may also act as a ligand for the cell surface receptor. These glycoproteins are involved in several cellular processes such as cell growth, cell-cell recognition, and cell migration, critical in angiogenesis. A possible alteration of the structure and therefore, their function was indicated by the identification of 56 DEGs that encode enzymes related to glycoprotein metabolism including 5 glycosyl transferases. *VEGF* receptor, a glycoprotein, may recognize other proteins in the absence of *VEGF* and triggers the downstream signaling. Furthermore, studies also reported that interaction of galectin-1 and *VEGFR2* activate *VEGF*-like signaling in tumor angiogenesis [56]. In this context, it is also pertinent to note that glioma cells employ different metabolic strategies including aerobic glycolysis, pentose phosphate pathway, one carbon metabolism, fatty acid metabolism which contribute to energy production in glioma cells and several bioenergetics pathways are linked to oncogenic signals such as *AMPK* and *MTOR* pathways [57,58].

One of the signaling pathways altered during the development of resistance to anti-*VEGF* therapy in GBM appears to be *BMP* signaling pathway. It is a complex network of receptors, ligands and antagonists which may dynamically impact GBM growth, maintenance and progression. *GREM1* is an antagonist of *BMP* signaling. Glioma stem cells secrete *GREM1* to promote tumorigenesis through inhibition of *BMP* signaling. Studies reported that the secretion of *GREM1* contribute to treatment resistance by maintaining cellular proliferation and cellular hierarchies within the tumor, and also increasing resistance to differentiation therapy [59]. The data presented here showed that gene encoding *GREM1* is upregulated about 3-fold, though *BMP*s and *TGFB* were slightly downregulated. However, certain other genes which are known to modulate angiogenesis did not show any significant change. For instance, *TSGA10* (testis specific Gene Antigen 10), which acts as a tumor suppressor in many types of human cancers [60] and inhibits *VEGF*-induced angiogenesis [61] was not differentially expressed in anti-*VEGF* resistant condition. Further, recent RNA seq analysis of anti-*VEGF* resistant ovarian cancer model showed upregulation of apelin/*APJ* receptor signaling pathway [62]. However, this receptor-ligand pair gene expression was not altered in anti-*VEGF* resistant glioma described in the present study, probably suggesting that mechanisms underlying anti *VEGF* resistance are different in different tumors.

Further analysis of DEGs in anti-*VEGF* resistant microarray data sets revealed that 19 ligand receptor pairs were differentially expressed. The receptors *CD44*, *F3*, *IL6ST*, *ITGB1*, *NRP2*, *PLAUR* and *EGFR* were upregulated. *CD44* is a trans-membrane glycoprotein receptor of hyaluronic acid which is overexpressed in GBM and enhances the GBM invasion, proliferation and therapy resistance [63]. It is also involved in epithelial-mesenchymal transition, angiogenesis, proliferation, invasion, and migration [64]. Genes encoding the ligands for *CD44* such as *SPP1*, *HBEGF* and *FGF2* were also upregulated. Another important signaling molecule involved in GBM is *EGFR* whose ligands such as *EREG*, *FGF13*, *HBEGF*, *TGFA* and *VEGFA* were also upregulated. *IL6* and *TFPI*, ligands of *F3* receptor were also upregulated. Four ligands of *IL6ST* and *ITGB1* receptors were upregulated and in the case of receptors *NRP2* and *PLAUR*, one ligand each was upregulated. These 12 ligands included factors with growth factor (10 genes) and cytokine activity (5 genes). In this context, earlier data on alteration in *NRP1* expression and activation of *TGFB* signaling restoring angiogenesis in anti-*VEGF* resistant GBM is particularly relevant [65]. Therefore, it appears that instead of the principal ligands of several of these receptors, the upregulated ligands were alternate ligands, suggesting development of alternate mechanisms for angiogenesis and tumor growth. In this context it is important to note that, though not all glioblastoma patients are resistant to anti-*VEGF* therapy, the possibility of angiogenesis-independent tumor progression by diffuse invasion of single tumor cell in brain, as reported recently [66].

PPI network was developed, including both up- and downregulated genes, to verify the interaction between these genes and how they are coordinately involved in the formation of resistance. Many genes with high connectivity in the PPI network were enriched in pathways in cancer, *PI3K-Akt* signaling pathway, Proteoglycans in cancer and *MAPK* signaling pathway. We have identified 21 hub genes with hybrid centrality score > 12, among which 10 were up- and 11 downregulated. The possible role of these hub genes in the development of anti *VEGF* resistance in GBM was suggested from the data showing interaction of 18 of these hub genes with 58 genes of different network modules in the *VEGF*- mediated angiogenesis signaling pathway [6,67]. In this context, our previous report on multiple phytochemicals of a poly-herbal formulation targeting multiple components of *VEGF*-*VEGFR2* pathway and inhibiting angiogenesis is particularly significant [68].

KEGG pathway analysis and GO enrichment analysis also demonstrated that these hub genes were associated with pathways in cancer and significantly involved in positive regulation of angiogenesis and negative regulation of apoptosis. *VEGF* pathway analyses revealed that nine upregulated hub genes (*IL6*, *EGFR*, *VEGFA*, *SRC*, *CXCL8*, *PTGS2*, *IDH1*, *APP* and *SQSTM1*) and five downregulated hub genes (*POLR2H*, *RPS3*, *UBA52*, *CCNB1* and *UBE2C*) are linked with several of the *VEGF* signaling pathway components. Across all the generations six (*IL6*, *CXCL8*, *PTGS2*, *IDH1*, *POLR2H*, *UBA52*) hub genes and in fourth and nine generation 11 (*IL6*, *CXCL8*, *PTGS2*, *IDH1*, *APP*, *SQSTM1*, *POLR2H*, *RSP3*, *UBA52*, *CCNB1*, *UBE2C*) hub genes were differentially expressed, and the maximum fold change was observed in fourth generation. Studies also reported that high expression of *IL6* [69], and *EGFR* [70], had worst survival outcome than low expression. Chang et al. reported that GBM patients with lower *IL6* expression showed longer survival time and a few patients with longer survival time did not show significant expression of *IL6*. [71]. *PTGS2* is another hub gene which was enhanced in radiation resistant glioma cells [27]. Ribosomal protein S3 is suggested to be a substrate for induction of radio- resistance in glioblastoma [72].

Further evidence linking these hub genes with the development of resistance, was provided by survival analysis which revealed that out of the 10 upregulated hub genes, expression of three genes (*VEGFA*, *CXCL8*, *IDH1*) was statistically significant and were associated with a worse prognosis among patients with GBM. However, expression levels of none of the downregulated hub genes, including six genes whose downregulation is known to relate with low survival and five genes whose downregulation is associated with longer survival [20], showed any statistically significant association with survival. Altered expression of these hub genes has been reported in GBM. *CXCL8* was upregulated in GBM compared to diffuse astrocytoma and its expression levels were positively associated with progression and poor prognosis of glioma [73]. As discussed before *VEGF* plays important role in angiogenesis and its expression is high in GBM patients compared to the healthy subjects [74]. Hub gene *IDH1* mutation in GBM patients showed a longer survival rate compared to the wild-type [75]. However, the potential role of these predicted hub genes need to be further examined experimentally. The lack of any clinically established biomarker in glioblastoma, unlike in other tumors makes it difficult to follow response to anti-angiogenic therapy and survival of malignant glioma. The expression of angiogenic target molecules and also patterns of tumor vascularization did not predict response to bevacizumab [76] highlighting the need for reliable predictive biomarkers. The results presented here predict that the hub genes associated with the GBM resistance to bevacizumab may be a potential therapeutic target or biomarker of anti-*VEGF* resistance of GBM.

## 5. Conclusions

We presume that these key hub genes identified by a series of bioinformatics analyses on DEGs between tumor samples and anti-*VEGF*-resistant samples are probably related to the sensitivity of glioblastomas to anti-*VEGF* therapy. These identified genes and their associated pathways provide a more detailed molecular mechanism of anti-*VEGF* resistance in GBM. However, further molecular and biological experiments are required to confirm the functions of the key hub genes in resistant GBM.

## Figures and Tables

**Table 1:** Common differentially expressed genes (DEGs) identified from the datasets.

DEGs	Gene Symbol
Upregulated	*ACSL3*, *ACTA2*, *ADGRG1*, *AKR1B1*, *ASAP2*, *ASS1*, *ATP2B4*, *BAALC*, *BHLHE40*, *CALD1*, *CCDC92*, *CLIC4*, *CSF3*, *CXCL8*, *FAM65A*, *FGF2*, *HIST2H2BE*, *HLA-B*, *HLA-DPA1*, *HLA-DRA*, *IDH1*, *IER3*, *IGFBP7*, *IL1B*, *IL6*, *INSIG1*, *KDELR3*, *LRRN3*, *MLLT11*, *MSMO1*, *MTSS1*, *NAMPT*, *NME4*, *NOTCH3*, *PAMR1*, *PDGFRB*, *PHKB*, *PPP1R3C*, *PTGS2*, *RCAN1*, *RFTN2*, *RHBDF1*, *RND3*, *RRAD*, *RSPO3*, *SEZ6L2*, *SH3BGRL*, *SMARCA1*, *SNX7*, *SPP1*, *SRPX2*, *SYNC*, *SYNDIG1*, *TAP1*, *TFPI2*, *TGM2*, *TMCO3*, *TMEM171*, *TMEM98*, *TOX2*, *TSPAN13*, *VAT1L*
Downregulated	*EIF6*, *NTSR1*, *IMP4*, *RPPH1*, *TOP3A*, *LYAR*, *THOC5*, *CXADR*, *FOXR2*, *DHRS11*, *DBNDD2*, *DDX27*, *DNAAF3*, *ALKBH2*, *IPO4*, *DUS3*, *MRPL12*, *PRPF19*, *INTS1*, *DDX18*, *FERMT3*, *EMG1*, *SNU13*, *SERPINB7*, *RPS19BP1*, *UBL7*, *RPS21*, *CCL26*, *SLC43A3*, *IL13RA2*, *NHP2*, *SNAPC4*, *GRWD1*, *PHB2*, *MRTO4*, *PDSS1*, *SPOUT1*, *DNLZ*, *GPATCH4*, *CCL20*, *EIF2B2*, *ZNF593*, *NOP16*, *NUDT14*, *MCM10*, *SLIRP*, *RRS1*, *KLRG1*, *ATF5*, *CDK4*, *COQ3*, *GEMIN4*, *GEMIN6*, *FTSJ3*, *PAK1IP1*, *MRPS26*, *NTS*, *TOMM22*, *EXOSC5*, *ATAD3A*, *GAR1*, *RPF2*, *TEAD4*, *PPAN*, *UBE2G2*, *C9orf142*, *PRPF4*, *PPIH*, *RPL36*, *TTLL12*, *WDR46*, *PSMG3*, *PRIM1*, *CSTF2*, *TOMM6*, *AHSA1*, *TFB2M*, *SLC5A6*, *NOP56*, *TIMM10*, *C10orf2*, *CLPTM1L*, *DPH2*, *C19orf48*, *FEN1*, *UBA52*, *FARSA*, *ELOF1*, *S100A2*, *NOL6*, *STOML2*, *ADSL*, *CHCHD10*, *POLR2H*, *PA2G4*, *E2F2*, *PRKAR1B*, *LBHD1*, *PRELID1*, *MRPL21*, *EXOSC4*, *TRIML2*, *NPM3*, *SNORD104*, *EBNA1BP2*, *WDR18*, *EIF3K*, *STC2*, *POLD1*, *SFXN4*, *SNORA67*, *METTL17*, *ATIC*, *EIF3G*, *SDCCAG3*, *MAGEB2*, *PNP*, *HCLS1*, *NDUFB9*, *DHRS2*, *CDC25A*, *ESM1*
Both up- and downregulated	*CRABP2*, *EDNRA*, *GJB2*, *IGFBP5*, *MATN2*, *MFAP4*, *NREP*, *OLFML2A*, *PCOLCE*, *PDGFRA*, *RGS2*, *SERPINB2*, *TGFB3*, *TIMP2*, *TRIM9*

Microarray data on anti-*VEGF*-resistant glioblastoma xenografts for the 1st, 4th, and 9th generations were downloaded from the Gene Expression Omnibus Database (GEO), and the DEGs were identified using GEO2R with a fold change (logFC) > 1 and logFC < −1. A total of 199 overlapped DEGs were identified, including 62 upregulated, 122 downregulated, and 15 genes showing both up- and downregulations from the 1st, 4th, and 9th generations, as described in detail in the legend to Figure 1.

**Table 2:** Classification of the DEGs.

DEGs Classification	Gene Symbol
Growth factors	*HBEGF*, *VEGFA*, *EGFR*, *PDGFRB*, *FGF13*, *FGF2*, *TFPI*, *TFPI2*, *TGFA*, *TGIF1*, *WNT5A*, *BDNF*, *NRG1*, *TIMP1*, *PDGFRA*, *TGFB3*
Cytokines	*CSF3*, *NAMPT*, *CCL3*, *IL6*, *CXCL2*, *CXCL8*, *GREM1*, *IL11*, *IL36RN*, *LIF*, *CLCF1*, *CCL3L3*, *IL36B*, *IL1B*, *IL1A*, *SPP1*, *IL1R1*, *NRG1*, *EREG*
*BMP*s	*BMP6*, *GREM1*, *NBL1*, *TWSG1*
Proto-oncogenes	*FYN*, *MLLT11*, *PDGFRB*, *BCL6*, *SRC*, *CBLB*, *CRKL*
Genes involved in GBM	*ESM1*, *PDGFRA*, *TGFB3*
Receptor–Ligand pairs	*CD44-SPP1*, *CD44-HBEGF*, *CD44-FGF2*, *F3-IL6*, *F3-TFPI*, *IL6ST-CLCF1*, *IL6ST-IL6*, *IL6ST-LIF*, *IL6ST-IL11*, *ITGB1-SPP1*, *ITGB1-THBS1*, *ITGB1-VEGFA*, *NRP2-VEGFA*, *PLAUR -SERPINE1*, *EGFR-EREG*, *EGFR-FGF13*, *EGFR-HBEGF*, *EGFR-TGFA*, *EGFR-VEGFA*
Enzymes (Glycosyl transferases)	*GBE1*, *PLOD2*, *HAS1*, *ST3GAL1*, *EDEM1*

DEGs identified from the 4th-generation xenografts were classified based on their functions and roles in tumor development and progression. The classifications and gene symbols are presented. GBM: glioblastoma multiforme and *BMPs*: Bone morphogenetic proteins.

**Table 3:** (KEGG pathway enrichment analysis of module 3 and module 4 of PPI network).

Term	Description	Count	*p*-Value	Gene Symbol
**Module 3**				
hsa04110	Cell cycle	9	6.28 × 10^−7^	*E2F2*, *CDKN1A*, *SKP2*, *MCM2*, *MCM3*, *CDK4*, *CDC25A*, *CDC25B*, *MCM6*
hsa04060	Cytokine–cytokine receptor interaction	7	0.003	*CXCL1*, *CCL3*, *CCL20*, *CXCL2*, *IL1B*, *IL1A*, *IL11*
hsa05206	MicroRNAs in cancer	7	0.007	*E2F2*, *CDKN1A*, *PTGS2*, *HMOX1*, *THBS1*, *CDC25A*, *CDC25B*
hsa04668	*TNF* signaling pathway	6	4.46 × 10^−4^	*CXCL1*, *PTGS2*, *CCL20*, *CXCL2*, *IL1B*, *MMP3*
hsa05200	Pathways in cancer	6	0.049	*E2F2*, *CDKN1A*, *HIF1A*, *PTGS2*, *SKP2*, *CDK4*
hsa05219	Bladder cancer	5	1.03 × 10^−4^	*E2F2*, *CDKN1A*, *THBS1*, *CDK4*, *SRC*
hsa04066	*HIF1* signaling pathway	4	0.020	*CDKN1A*, *HIF1A*, *HMOX1*, *SERPINE1*
hsa04621	*NOD*-like receptor signaling pathway	3	0.045	*CXCL1*, *CXCL2*, *IL1B*
hsa05214	Glioma	3	0.049	*E2F2*, *CDKN1A*, *CDK4*
**Module 4**				
hsa05200	Pathways in cancer	6	0.002	*VEGFA*, *TGFA*, *PDGFRB*, *JAK1*, *FGF13*, *FGF2*
hsa04151	*PI3K-Akt* signaling pathway	5	0.008	*VEGFA*, *PDGFRB*, *JAK1*, *FGF13*, *FGF2*
hsa05212	Pancreatic cancer	3	0.011	*VEGFA*, *TGFA*, *JAK1*
hsa05205	Proteoglycans in cancer	4	0.012	*VEGFA*, *HBEGF*, *SDC4*, *FGF2*
hsa05218	Melanoma	3	0.013	*PDGFRB*, *FGF13*, *FGF2*
hsa04015	*Rap1* signaling pathway	4	0.014	*VEGFA*, *PDGFRB*, *FGF13*, *FGF2*
hsa04014	*Ras* signaling pathway	4	0.017	*VEGFA*, *PDGFRB*, *FGF13*, *FGF2*
hsa04010	*MAPK* signaling pathway	4	0.023	*DUSP1*, *PDGFRB*, *FGF13*, *FGF2*
hsa05219	Bladder cancer	2	0.038	*VEGFA*, *HBEGF*

Modules from the PPI network were extracted using the MCODE plugin in Cytoscape, and scores > 5 were subjected to an enrichment analysis. An enrichment analysis was done using the Database for Annotation, Visualization and Integrated Discovery (DAVID), and a set count >2 and *p* < 0.05 as the cut-off for significant enrichment. A total of 7 modules were identified using the MCODE plugin in Cytoscape—out of which, 2 modules (modules 3 and 4) are functionally relevant. List of genes enriched in different pathways in module 3 and 4 is presented.

**Table 4:** Identification of up- and downregulated hub genes among the DEGs.

DEGs	Gene Symbol
Upregulated	*IL6*, *VEGFA*, *SRC*, *APP*, *CXCL8*, *IDH1*, *SQSTM1*, *EGFR*, *PTGS2*, *ALDOA*
Downregulated	*NCL*, *RPS3*, *UBA52*, *DNMT1*, *CCNB1*, *EZH2*, *PLK1*, *POLR2H*, *UBE2C*, *TYMS*, *PCNA*

Hub genes were identified using a hybrid centrality measure method. Out of 792 nodes, 21 hub genes were identified with a hybrid centrality score> 12 and classified into upregulated and downregulated hub genes based on the logFC values. Ten upregulated and 11 downregulated hub genes were represented using gene symbols.

**Table 5:** Pathway enrichment analysis of the hub genes.

Table	Description	Count	*p*-Value	Gene Symbol
hsa05219	Bladder cancer	4	0.0002	*CXCL8*, *SRC*, *EGFR*, *VEGFA*
hsa04068	*FoxO* signaling pathway	4	0.006	*IL6*, *CCNB1*, *PLK1*, *EGFR*
hsa05206	MicroRNAs in cancer	5	0.008	*DNMT1*, *PTGS2*, *EGFR*, *EZH2*, *VEGFA*
hsa04370	*VEGF* signaling pathway	3	0.013	*SRC*, *PTGS2*, *VEGFA*
hsa05120	Epithelial cell signaling in Helicobacter pylori infection	3	0.016	*CXCL8*, *SRC*, *EGFR*
hsa05200	Pathways in cancer	5	0.024	*IL6*, *CXCL8*, *PTGS2*, *EGFR*, *VEGFA*
hsa04066	*HIF1* signaling pathway	3	0.031	*IL6*, *EGFR*, *VEGFA*
hsa04110	Cell cycle	3	0.049	*CCNB1*, *PCNA*, *PLK1*

Enrichment analysis was done using DAVID and a set count >2 and *p* < 0.05 as the cut-off for significant enrichment. List of hub genes enriched in different pathways are presented.

**Table 6:** Identification of the hub genes related to the survival of GBM patients.

Upregulated Hub Genes	Survival Rate (in Months)	Log-Rank *p*	*p*(HR)	Downregulated Hub Genes	Survival Rate (in Months)	Log-Rank *p*	*p* (HR)
*IL6*	49	0.07	0.074	*PCNA*	52	0.87	0.85
*VEGFA*	49	0.05	0.046	*POLR2H*	52	0.46	0.47
*SRC*	52	0.24	0.25	*RPS3*	52	0.07	0.077
*CXCL8*	49	0.04	0.05	*UBA52*	52	0.14	0.15
*IDH1*	49	0.03	0.029	*CCNB1*	71	0.7	0.72
*PTGS2*	52	0.54	0.54	*UBE2C*	71	0.77	0.79
*EGFR*	49	0.93	0.9				
*APP*	71	0.46	0.47				
*ALDOA*	71	0.11	0.11				
*SQSTM1*	71	0.13	0.13				

Analysis was done using the Gene Expression Profiling Interactive Analysis (GEPIA) online tool, and the relationship between the hub gene expression and its significance in resistance was verified using the method of Kaplan–Meier for the survival analysis, as described in detail in the legend to Figure 6. The survival rate, log-rank *p*, and hazard ratio (HR) of the hub genes were extracted from Figure 6 and presented. *p* and *p*(HR) ≤ 0.05 were considered significant.

## Supplementary Materials

The following are available online at https://www.mdpi.com/2218-273X/11/3/403/s1, Figure S1: Common biological process enrichment in up-regulated DEGs in 4th and 9th generations, Figure S2: KEGG pathway enrichment analysis of downregulated DEGs, Figure S3: Analysis of the protein–protein interaction network of DEGs, Figure S4: Survival analysis of 5 down-regulated hub genes in patients with GBM. Table S1: The number of biological processes, pathways and genes enriched in 1st, 4th and 9th generation, Table S2: Classification of DEG’s, Table S3: Identification of significant modules from PPI network, Table S4: KEGG pathway enrichment analysis of module 2 and module 4 of 1st generation PPI network, Table S5: KEGG pathway enrichment analysis of module 4, 6 and 9 of 9th generation PPI network, Table S6: Identification of up- and down-regulated hub genes among 1st generation DEGs, Table S7: Identification of up- and down-regulated hub genes among 9th generation DEGs, Table S8: Pathway enrichment analysis of 1st generation hub genes, Table S9: Pathway enrichment analysis of 9th generation hub genes, Table S10: Hub genes and VEGF pathway association.
