# Supplementary material for: Identification of Hub Genes and Key Pathways Associated with Anti-VEGF Resistant Glioblastoma Using Gene Expression Data Analysis
Source: Biomolecules. 2021 Mar 9;11(3):403. doi: 10.3390/biom11030403 (PMC8000064; doi:10.3390/biom11030403)
Supplement: Supplementary file 1 [file biomolecules-11-00403-s001.zip › biomolecules-1131332-supplementary/Supplementary Figures.pdf]

Supplementary Figure S1: Common biological process enrichment in up-regulated DEGs in 4th and 9th generations

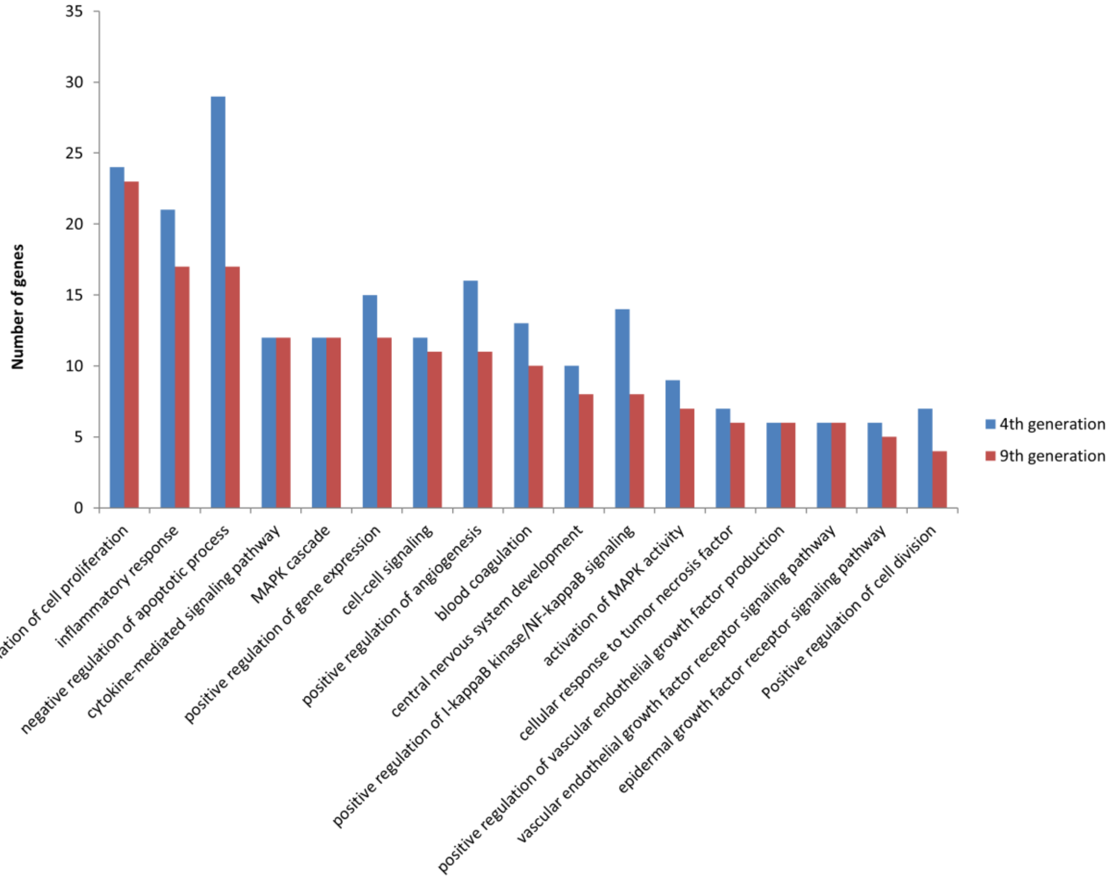

The differentially expressed genes identified were subjected to enrichment analysis using DAVID and set count > 2 and $p < 0.05$ as the cut off for significant enrichment. The number of DEGs enriched for major biological processes related to cancer and angiogenesis are represented.

Supplementary Figure S2: KEGG pathway enrichment analysis of down-regulated DEGs

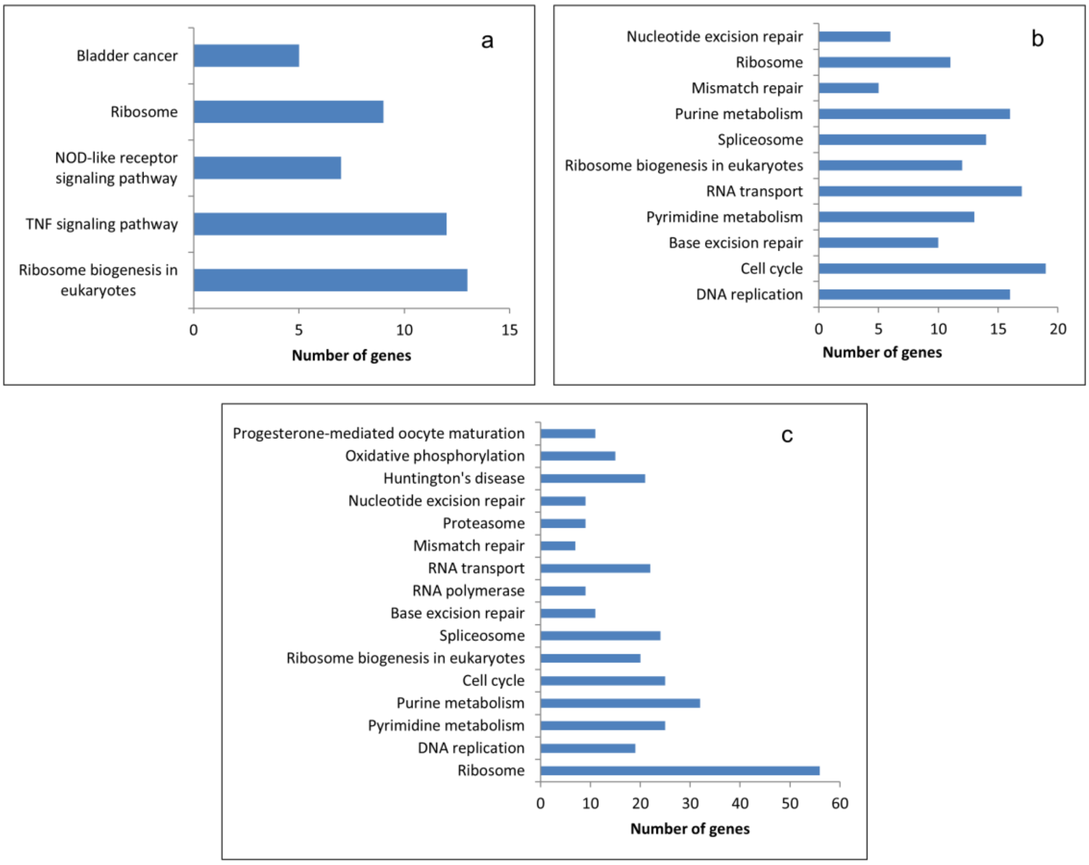

The DEGs identified were subjected to pathway enrichment analysis using DAVID and set count > 2 and $p < 0.05$ as the cut off for significant enrichment. The enriched pathways related to cancer and angiogenesis a) 1st generation b) 4th generation c) 9th generation are represented.

Supplementary Figure S3: Analysis of protein-protein interaction network of DEGs

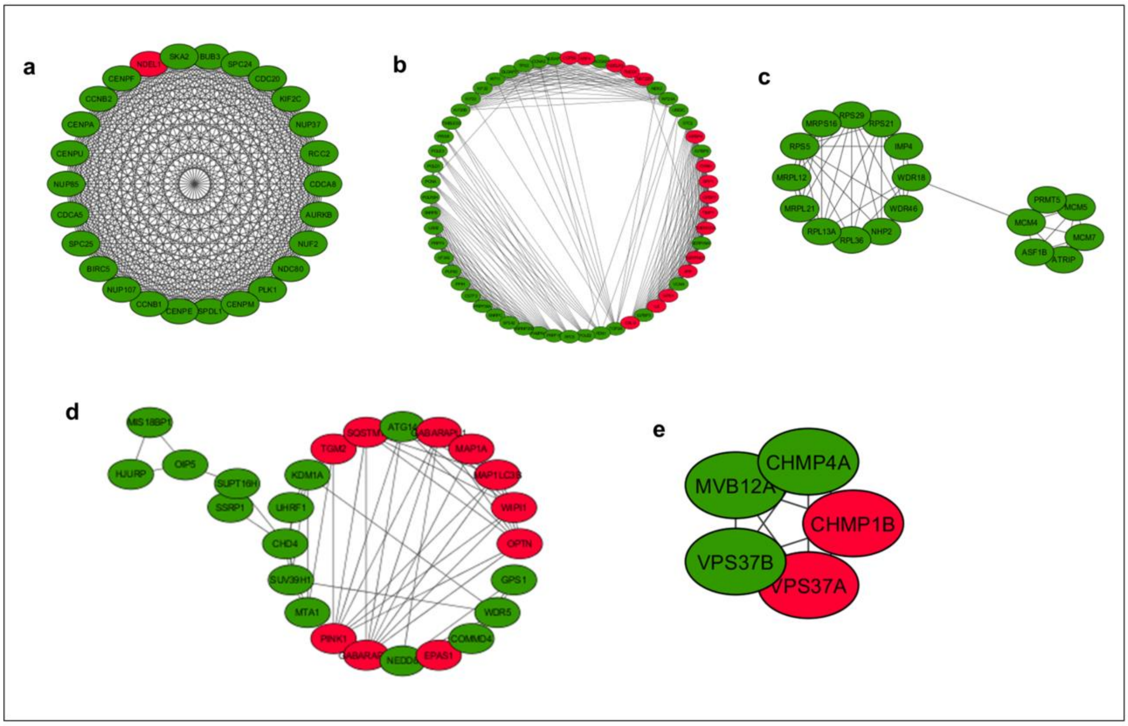

PPI network of DEGs were developed by STRING and analysed using Cytoscape. Modules from the PPI were extracted using MCODE plugin in Cytoscape with default thresholds, degree cut off: 2, node score cut off: 0.2, k-core: 2, and max depth: 100. Seven modules (4th generation) with node score > 5 considered to be significant. (a) Module 1 with 26 nodes and 325 edges (b) Module 2 with 56 nodes and 352 edges (c) Module 5 with 18 nodes and 51 edges (d) Module 6 with 25 nodes and 64 edges (e) Module 7 with 5 nodes and 10 edges are represented. Up-regulated genes are marked in red; and down-regulated ones in green. Module 3 and Module 4 are included in main text.

Supplementary Figure S4. Survival analysis of 5 down-regulated hub genes in patients with GBM.

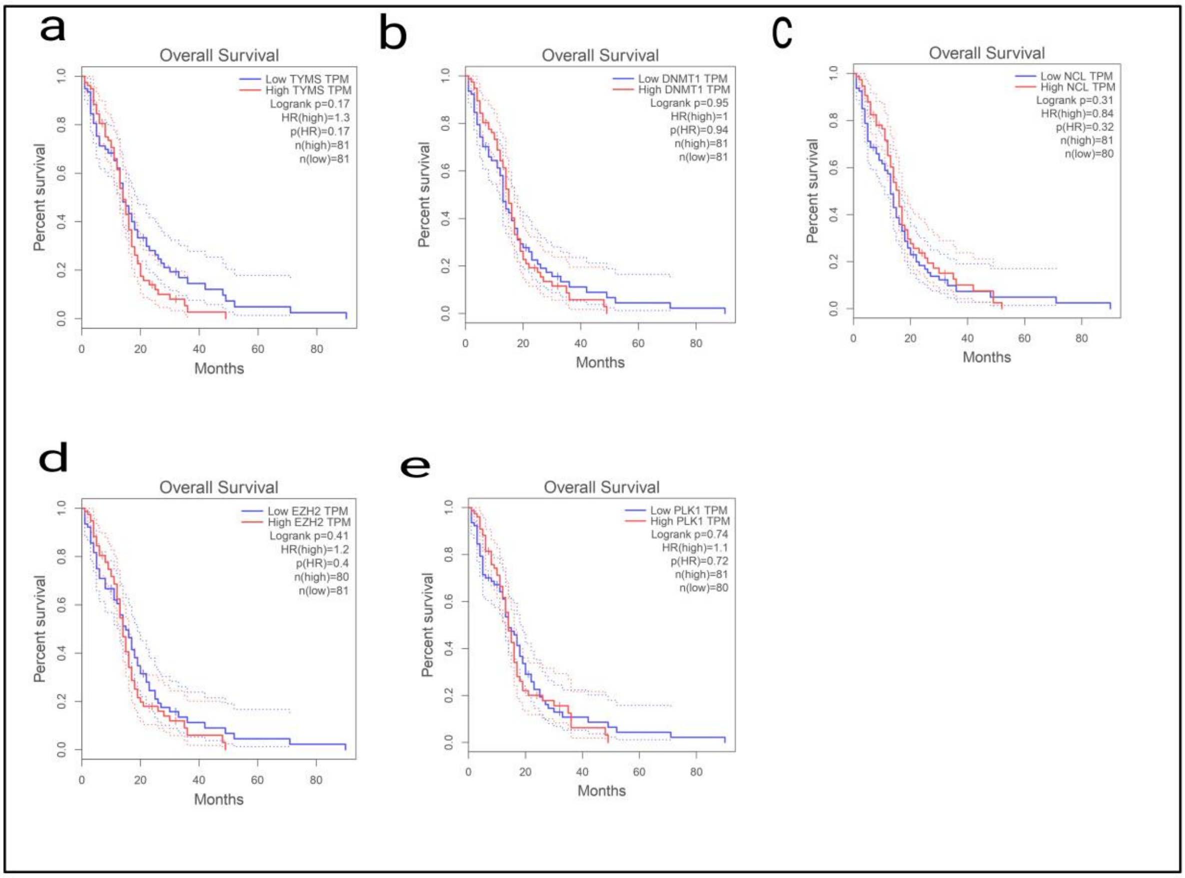

The relationship between the expression of hub genes and survival as analyzed by plotting high and low expression levels of up- and down-regulated hub genes in patients with GBM. The survival curves were plotted using GEPIA. The specific DEGs expression levels were dichotomized by median value. The results are presented visually by Kaplan-Meier survival plots. p-values were calculated using log-rank statistics. GBM, glioblastoma; HR, hazard ratio; TPM, transcripts per million. survival plot of down-regulated hub genes (a) *TYMS* (b) *DNMT1* (c) *NCL* (d) *EZH2* (e) *PLK1*.
