# Supplementary material for: Identification of Hub Genes and Key Pathways Associated with Anti-VEGF Resistant Glioblastoma Using Gene Expression Data Analysis
Source: Biomolecules. 2021 Mar 9;11(3):403. doi: 10.3390/biom11030403 (PMC8000064; doi:10.3390/biom11030403)
Supplement: Supplementary file 1 [file biomolecules-11-00403-s001.zip › biomolecules-1131332-supplementary/Supplementary Tables .pdf]

Supplementary Table S1: The number of biological processes, pathways and genes enriched in 1st, 4th and 9th generation

	1st generation	4th generation	9th generation
Number of gene enriched	275	368	271
Number of Biological processes enriched	73	127	92
Number of pathways enriched	16	27	10

The differentially expressed genes identified were subjected to enrichment analysis using DAVID and set count > 2 and $p < 0.05$ as the cut off for significant enrichment. The number of biological processes, pathways and number of genes enriched therein are represented.

Supplementary Table S2: Classification of DEG's

Term	Gene symbol
Membrane associated protein	HLA-B, ITGAX, CRELD1, DENND5A, LITAF, PTPRN, SGCE, SLC39A14, SPRY4, CYP4B1, GFPT2, HSD17B12, MGST1, RHBDF1, KLHL5, MYOF, NOMO1, PFN2, ATP1B1, BCAP29, CLDND1, TMBIM1, TMCO3, DNER, TMEM98, BDKRB1, HLA-DRA, INSIG1, RTN1, SLC25A43, SLC35G2, SYNDIG1, VASN, TNFRSF12A, ACSL3, HERC4, PTPRR, ATP2B4, HAS1, PAM, GJA1, SPRY2, ATP2B1, MAN1A1, NRP2, PLAUR, RRM2B, VKORC1, NOTCH3, ENPP2, CYP1B1, GFPT1, PLOD2, PNPLA8, CREB3L2, XBP1, NRG1, ITGA2, MSMO1, STT3B, SLC33A1, TGFA, TPM1, ACVR1, HMOX1, NT5E, ADORA2A, APP, BDNF, ATP2C1, WFS1, IL6ST, ITGB1, TAP1, PRNP, ATP2A2, CD44, F3, ABCC2, ASPH, PDGFRB, PMEPA1, CA9, PCSK7, NRDE2, PCDH7, POLR1E, ZNF511, ABCB9, SLC18B1, SLC43A3, STEAP3, SURF1, UQCC3, RHBDD2, ADAM19, IFITM2, LAPTM4B, OXA1L, SLC25A51, SLC35C2, SYNJ2BP, TMEM97, CD70, ADCY3, PARL, TRPM8, ATP13A1, SLC5A6, IL13RA2, CD320, IFNGR2, NTSR1, SDC4, TAF15, DHCR24, SFXN4, SLC25A15, SLC25A19, EDNRA, COQ2, GJB2, OCA2, QARS, VRK1, AXL, CSF2RA, EPHB2, PDGFRA
Secretary protein	IL11, IL36RN, SRPX2, PAMR1, SCPEP1, VASN, CCL3, RSPO3, CLCF1, GREM1, SMOC1, IGFBP4, IGFBP6, PAM, LIF, QPCT, NRP2, CSF3, CXCL2, SPP1, IGFBP7, WNT5A, NRG1, TIMP1, NAMPT, IL1A, TGFA, CXCL8, THBS1, TFPI, BDNF, HTRA1, IL6ST, PAPPA, SERPIND1, LOX, CD44, F3, IL6, IL1B, FGF2, SERPINE1, MMP3, VEGFA, COL7A1, THBS3, CLEC11A, IGFBP5, MFAP4, PCOLCE, SERPINE2, TNFAIP6, ADM, IGFBP3, VCAN, CPA4, PRSS3, SDC4, CXCL1, TGFB3, NOV, SERPINA3, TIMP2, SERPINA1, COL1A1, CSF2RA
Intracellular protein	C18orf8, CDKN1A, GCLM, PHLDA1, CYP26B1, DOCK7, HSPB3, HSPB8, KIAA0556, KIF7, KLHL7, OPTN, OTOGL, PHKB, SEC24D, SQSTM1, TRAF3IP2, VPS37A, WBP2, CHST7, GALNT5, IRAK2, NMNAT2, NMRK1, PTPN12, RIPK2, ST3GAL1, TGM2, TRIM5, UBE2H, TUBE1, ARF4, ARHGDIB, CALD1, CALU, CDH13, CLIP2, COPB2, DAB2, DNAJB4, EDEM1, FGD3, GLRX, MORF4L2, MTSS1, RAB27B, RGS2, SH3BGRL, SRGN, TAGLN3, UBQLN1, UFM1, VAT1L, GNG11, ATF3, BHLHE40, EBF2, MAFF, NFKBIZ, SMARCA1, TOX2, ZNF175, AP1G1, CLIC4, SH3GLB1, SLC25A24, HLA-B, ITGAX, CRELD1, DENND5A, LITAF, PTPRN, SGCE, SLC39A14, SPRY4, CYP4B1, GFPT2, HSD17B12, MGST1, RHBDF1, KLHL5, MYOF, NOMO1, PFN2, ATP1B1, BCAP29, CLDND1, TMBIM1, TMCO3, IL11, IL36RN, SRPX2, PAMR1, SCPEP1, MLLT11, SH2B3, BIRC2, DUSP1, MAGEC1, RBL2, ACTA2, CITED2, IRS1, MCFD2, PLS3, PRKAG2, VPS35, DDAH1, FYN, HAGH, NME4, P4HA1, PIR, MAP1A, NKX3-1, EGR2, IRX5, MEIS2, SNAI2, NFE2L1, SDCBP, TNFRSF12A, ACSL3, HERC4, PTPRR, ATP2B4, HAS1, PAM, LIF, QPCT, PTGS2, PRKAR1A, AKR1B1, SERPINB2, ALDH1A3, FKBP14, IDS, OAT, P3H2,

	PAPSS2, PGM3, PINK1, PREPL, SAT1, BCL6, DDIT3, TGIF1, NR4A2, HIF1A, PLAUR, RRM2B, VKORC1, NOTCH3, ENPP2, CYP1B1, GFPT1, PLOD2, PNPLA8, CREB3L2, XBP1, NRG1, TIMP1, NAMPT, IL1A, JAK1, TXNRD1, ASS1, DUSP6, GBE1, HSD17B4, KYNU, PTPN22, UCHL1, EPAS1, FLNC, TPM1, ACVR1, HMOX1, NT5E, ADORA2A, APP, BDNF, HTRA1, ALDOA, RARB, ITGB1, TAP1, PRNP, ATP2A2, CD44, F3, IL6, IL1B, FGF2, FTH1, IDH1, SRC, ASPH, MMP3, VEGFA, PDGFRB, AHNAK2, BIRC5, CCNA2, GPS2, S100A2, SKP2, STMN1, TPX2, ATAD3A, BIN1, CDCA7, CDT1, CEP78, CHCHD10, D2HGDH, EIF2B1, EIF2B2, GLRX5, HIST1H4C, KDM1A, PUF60, RNASEH2C, SERPINB7, TDP1, TRIP13, WDR34, ADPRHL2, ALKBH2, C14orf169, COQ3, DDX27, DDX39A, DHRS11, DHRS4, DPH2, ELP3, FARSA, GMDS, GNPNAT1, IMPA2, MKNK2, NEURL1B, NUDT14, PAFAH1B3, PPCS, PRMT6, RNF31, RPP40, SETMAR, SULT1A1, SUV39H1, TRIM9, TRIML2, TRMT61A, TSTA3, AHSA1, CIRBP, CSTF2, DLGAP5, EIF2S1, EIF3G, EIF6, EXOSC4, FBL, GAR1, GEMIN4, GPS1, HAUS7, HCLS1, HMGB2, KHSRP, KIF15, KIFC1, KRT80, LSM2, NASP, NCAPD2, NCL, NUCKS1, NUF2, PHB2, PPAN, PRELID1, PSME1, RANBP1, RCC1, RFC5, SERBP1, SF3A2, SF3A3, SHROOM2, SNRPC, TLN2, TTLL12, TUBGCP2, WDR18, WDR46, MRPL12, MRPL21, MRPL52, MRPS26, MRTO4, RPL36, RPL39L, RSL1D1, UBA52, POLR2H, POLR3G, ATF5, FOXR2, FOXS1, LYAR, SNAPC4, TEAD4, ZNF581, ZNF786, ATG14, CLNS1A, EMC9, EXOC3, IPO4, NUP37, NUP85, PNN, PMEPA1, CA9, PCSK7, NRDE2, PCDH7, POLR1E, ZNF511, ABCB9, SLC18B1, SLC43A3, COL7A1, THBS3, CDC20, EZR, FEN1, FOXM1, ILF3, KIF2C, NEDD8, NFIB, NUDT1, RBBP4, SERPINB4, TOP3A, UBE2C, CENPE, DKC1, EMG1, FERMT3, FOXG1, HMGB3, KIF11, KIF22, LMNB2, MRPS16, NHP2, NOP56, PABPN1, PRDM8, PRPF4, RPS29, SNRPB, TFAM, APEH, CPE, DDX18, DDX24, GMPR2, LIG1, MAT2A, MCM6, METTL3, OTUB1, PA2G4, PARP2, PBK, POLE2, POLE3, PPIH, PRMT5, PRPF19, RPS6KA4, SHMT2, SUPT16H, TOP1MT, TRIM28, UBE2G2, USP13, TUBB4B, RORB, ACIN1, E2F2, RPL13A, RPS21, RPS5, SMARCC1, SSRP1, CD70, ADCY3, PARL, TRPM8, ATP13A1, SLC5A6, ADM, IGFBP3, VCAN, CPA4, PRSS3, ASPM, CDC25A, CDC25B, CENPF, MCM3, MCM7, PCNA, SMARCB1, TK1, U2AF1, IMPDH2, ADSL, C10orf2, CDKN3, COASY, EIF2AK4, GPD1L, ITPA, MCM4, NDUFV1, NEK2, PADI3, PDSS1, PMVK, PNPT1, RNASEH2A, SARS2, TRMT5, TSEN2, UHRF1, UNG, MTA1, CCNB1, CCNB2, FABP5, NUP107, TAF15, DHCR24, SFXN4, SLC25A15, SLC25A19, SERPINA3, TIMP2, APEX1, PARP1, TOP2A, TYMS, PNP, PLK1, POLQ, PYGL, RPIA, SNRNP200, RPL5, RPS3, HBA2, QARS, VRK1, SERPINA1, COL1A1, ATIC, CDK4, AURKB, CAD, CHD4, CHD8, EZH2, MCM2, MCM5, POLD1, ENO3, DNMT1, NUP214, EPHB2, PDGFRA
**Transcription factor**	ATF3, BHLHE40, EBF2, MAFF, NFKBIZ, SMARCA1, TOX2, ZNF175, NKX3-1, EGR2, IRX5, MEIS2, SNAI2, NFE2L1, BCL6, CREB3L2, DDIT3, TGIF1, XBP1, NR4A2, HIF1A, EPAS1, RARB, ATF5, FOXR2, FOXS1, LYAR, SNAPC4, TEAD4, ZNF511, ZNF581, ZNF786, E2F2, FOXG1, FOXM1, NFIB, PRDM8, RORB, SMARCC1, SSRP1, TFAM, DNMT1, MTA1
**Glycoprotein**	SCPEP1, ATP1B1, PTGS2, IL6ST, WFS1, MMP3, DNASE1L1, ST3GAL1, SEZ6L2, BDNF, APP, CD44, IDS, PLOD2, PAPPA, SERPINE1, CREB3L2, LOX, ASPH, TFPI2, IL1A, ITFG1, SLC33A1, PTPRR, TMEM132A, HLA-B, PTPRN, PLAUR, PNPLA8, F3, VEGFA, TFPI, PDGFRB, HLA-DPA1, STC1, FAM171A1, HLA-DRA, WNT5A, TWSG1, PAM, CRELD1, ENPP2, ADORA2A, PAMR1, ACP7, BDKRB1, GREM1, ITGB1, CALU, TIMP1, P3H2, LIF, ITGAX, SRGN, SPP1, ADAM23, UST, RHBDF1, ITGA2, OTOGL, ADGRG1, NOTCH3, SRPX2, NOMO1, NRP2, IER3, IGFBP7, IGFBP6, UCHL1, CLCF1, ITPRIP, P4HA1, RSPO3, TGFA, CLDND1, NRG1, NT5E, LARGE1, VASN, LY96, MAN1A1, EREG, CHST7, HIST2H2BE, FKBP14, SERPINB2, SGCE, TM4SF1, TMCO3, PRNP, ACVR1, CSF3, SLC39A14, EPDR1, GALNT5, EDEM1, PLPP3, STT3B, TMED7, SMOC1, DNER, POPDC3, THBS1, FAM174A, IL6, NCEH1, SVEP1, LRRN3, TSPAN13, QPCT, CDH13, HBEGF, SERPIND1, ABCC2, IGFBP4

DEGs identified from the 4th generation xenografts were classified based on their functions and role in tumor development and progression. The classifications and gene symbols are presented.

Supplementary Table S3: Identification of significant modules from PPI network

Module	Node Score	Nodes	Edges	Node IDs
1	26	26	325	SPC24, BIRC5, NDEL1, CENPE, CENPF, AURKB, CDCA8, RCC2, CCNB2, CDC20, SPDL1, SPC25, CENPA, KIF2C, NUP107, NUP85, CDCA5, NDC80, NUF2, CCNB1, SKA2, CENPM, NUP37, BUB3, CENPU, PLK1
2	12.8	56	352	CALU, STC2, KIF11, IGFBP7, NEK2, CYR61, UBE2C, TIMP1, CCNA2, SEC22B, IGFBP5, KIF22, SERPIND1, DLGAP5, IGFBP3, TMED7, TMEM132A, SNRPC, PUF60, PRPF19, SNRPB, IL6, ARF4, PRIM1, COPB2, APP, LSM2, TIMELESS, POLE2, PABPN1, POLE3, CSTF2, KDELR3, POLR2H, PRPF38A, KIF20B, FEN1, POLD1, RFC5, IGFBP4, SPP1, PCNA, TOP2A, PPIH, WFS1, TPX2, KIF23, RACGAP1, SF3A2, SF3A3, VCAN, KIF20A, NUSAP1, SNRNP200, SERPINA1, PRPF4
3	9.683	64	305	LOX, NOL6, MCM2, MCM10, LRR1, LIG1, CDK4, BOP1, IL11, MMP3, EIF3K, EIF3G, SERPINE1, FBXL13, CXCL2, EIF2S1, PARP1, TRIM9, THBS1, UBE2H, CCL20, CXCL1, APEX1, SKP2, MAK16, MRTO4, IL1A, IL1B, HIF1A, CDC25B, PARP2, EXOSC4, CDT1, RPS3, EXOSC5, EXOSC9, UBE2G2, EBNA1BP2, FTSJ3, KLHL5, FBL, RPP40, EIF6, EXO1, HMOX1, TOP3A, RSL1D1, MCM3, TIMP2, MCM6, E2F2, PTGS2, CDKN1A, RPL39L, SRC, NHP2L1, EMG1, CDC25A, HERC4, CCL3, RRS1, RPF2, UBAC1, FOXM1
4	6.182	23	68	QPCT, TRAPPC6B, F3, FGF13, COL7A1, SEC24D, FGF2, PRSS3, HBEGF, DUSP1, SDC4, TGFA, FTH1, PDGFRB, TNFAIP6, MCFD2, BET1, USO1, IDH1, NT5E, VEGFA, JAK1, GJA1
5	6	18	51	RPL13A, ATRIP, RPS5, MRPL12, RPS21, MRPL21, MRPS16, PRMT5, MCM5, WDR18, RPS29, NHP2, WDR46, MCM4, MCM7, IMP4, RPL36, ASF1B
6	5.333	25	64	UHRF1, MTA1, MAP1A, GPS1, TGM2, WDR5, HJURP, ATG14, NEDD8, SUV39H1, KDM1A, PINK1, GABARAPL1, GABARAPL2, COMMD4, OIP5, MIS18BP1, MAP1LC3B, SQSTM1, WIPI1, CHD4, SUPT16H, EPAS1, SSRP1, OPTN
7	5	5	10	VPS37A, VPS37B, CHMP4A, MVB12A, CHMP1B

PPI network of DEGs were developed by STRING and analysed using Cytoscape. Modules from the PPI were extracted using MCODE plugin in Cytoscape with default thresholds, degree cut off: 2, node score cut off: 0.2, k-core: 2, and max depth: 100. Seven modules with node score > 5 considered to be significant. List of genes in each module with its node score and number of nodes and edges are presented.

Supplementary Table S4: KEGG pathway enrichment analysis of module 2 and module 4 of 1st generation PPI network

Term	Description	Count	P-value	Gene symbol
**Module 2**				
**hsa04512**	ECM-receptor interaction	9	2.94E-09	COL3A1, COL6A3, COL1A2, COL6A1, COL1A1, COL5A2, COL5A1, COL4A6, COL4A5
**hsa04514**	Cell adhesion molecules (CAMs)	10	7.23E-09	HLA-DRB1, HLA-A, HLA-DRB5, HLA-DPA1, HLA-B, HLA-DMB, HLA-DMA, HLA-G, HLA-DRA, HLA-F
**hsa04510**	Focal adhesion	9	2.46E-06	COL3A1, COL6A3, COL1A2, COL6A1, COL1A1, COL5A2, COL5A1, COL4A6, COL4A5
**hsa04151**	PI3K-Akt signaling pathway	9	1.05E-04	COL3A1, COL6A3, COL1A2, COL6A1, COL1A1, COL5A2, COL5A1, COL4A6, COL4A5
**Module 4**				
**hsa04060**	Cytokine-cytokine receptor interaction	4	0.002	CSF3, TNFRSF1B, CCL20, CXCL2
**hsa04668**	TNF signaling pathway	3	0.006	TNFRSF1B, CCL20, CXCL2

Modules form PPI network were extracted using MCODE plugin in Cytoscape and score > 5 were subjected to enrichment analysis. Enrichment analysis was done using DAVID and set count >2 and p < 0.05 as the cut off for significant enrichment. A total of 6 modules were identified using MCODE plugin in Cytoscape out of which 2 modules (module 2 and 4) are functionally significant. List of genes enriched in different pathways in module 2 and 4 are presented

Supplementary Table S5. KEGG pathway enrichment analysis of module 4 ,6 and 9 of 9th generation PPI network

Term	Description	Count	P-value	Gene symbol
**Module 4**				
**hsa04110**	Cell cycle	4	0.014	CCND1, SKP2, ANAPC11, CDC25A
**hsa05222**	Small cell lung cancer	3	0.049	CKS1B, CCND1, SKP2
**Module 6**				
**hsa04110**	Cell cycle	8	5.22E-09	E2F2, MCM2, MCM3, MAD2L2, CDK4, MCM4, MCM5, MCM6
**hsa05222**	Small cell lung cancer	3	0.016	E2F2, CKS2, CDK4
**Module 9**				
**hsa01100**	Metabolic pathways	12	0.004	NME4, PRIM1, ITPA, POLR1E, NME1-NME2, NME1, POLR1D, POLD2, NTPCR, ZNRD1, POLA2, POLR3C
**hsa04110**	Cell cycle	4	0.012	CDC45, PTTG1, CCNA2, CDC25B

Modules form PPI network were extracted using MCODE plugin in Cytoscape and score > 5 were subjected to enrichment analysis. Enrichment analysis was done using DAVID and set count >2 and $p < 0.05$ as the cut off for significant enrichment. A total of 11 modules were identified out of which 3 modules (module 4, 6 and 9) are functionally significant. List of genes enriched in different pathways in module 4, 6 and 9 are presented.

Supplementary Table S6: Identification of up- and down-regulated hub genes among 1st generation DEGs

DEGs	Gene symbol
Upregulated	ACTA2,PRKCA,COL1A1,GJA1,APOE,B2M,IDH1,BMPR2,ATP2A1,SOCS1,TGM2,CLIP3
Downregulated	IL6,UBA52,FGF2,CXCL8,IL1B,TFRC,RPL6,HSPA4,ADORA2B,SPP1,WDR18,CCT2,RPL36,POLD1,DPM2,PTGS2,NHP2L1,ITGB5,POLR2H,RPL34,CDKN1A,PRKCZ,EXOSC4,RPL23A,MMP3

Hub genes were identified using Hybrid centrality measure method. Out of 553 nodes 37 hub genes were identified with Hybrid centrality score> 12 and classified them into upregulated and downregulated hub genes based on logFC values. Twelve upregulated and 25 downregulated hub genes were represented using gene symbols.

Supplementary Table S7: Identification of up- and down-regulated hub genes among 9th generation DEGs

DEGs	Gene symbol
Upregulated	IL6,APP,CXCL8,HSPA5,SOD2,HIST2H2BE
Downregulated	GAPDH,UBA52,RPS27A,ACTB,CDK1,CCND1,NHP2L1,PLK1,RPS3,RPS6

Hub genes were identified using Hybrid centrality measure method. Out of 1134 nodes 16 hub genes were identified with Hybrid centrality score> 14 and classified them into upregulated and downregulated hub genes based on logFC values. Six upregulated and 10 downregulated hub genes were represented using gene symbols.

Supplementary Table S8: Pathway enrichment analysis of 1st generation hub genes

Term	Description	Count	P-value	Genes
**hsa04151**	PI3K-Akt signaling pathway	7	0.004	COL1A1 , IL6, CDKN1A, ITGB5, SPP1, PRKCA, FGF2
**hsa04066**	HIF-1 signaling pathway	4	0.009	IL6, CDKN1A, TFRC, PRKCA
**hsa04620**	Toll-like receptor signaling pathway	4	0.012	IL6, CXCL8, IL1B, SPP1
**hsa04668**	TNF signaling pathway	4	0.013	IL6, IL1B, MMP3, PTGS2
**hsa04621**	NOD-like receptor signaling pathway	3	0.027	IL6, CXCL8, IL1B
**hsa05200**	Pathways in cancer	6	0.033	IL6, CDKN1A, CXCL8, PRKCA, PTGS2, FGF2
**hsa05202**	Transcriptional misregulation in cancer	4	0.041	IL6, CDKN1A, CXCL8, MMP3
**hsa05206**	MicroRNAs in cancer	5	0.042	CDKN1A, BMPR2, SOCS1, PRKCA, PTGS2

Enrichment analysis was done using DAVID and set count >2 and $p < 0.05$ as the cut off for significant enrichment. List of hub genes enriched in different pathways are presented. Up-regulated genes are marked in red; and down-regulated ones in black.

Supplementary Table S9: Pathway enrichment analysis of 9th generation hub genes

Term	Description	Count	P-value	Genes
**hsa04068**	FoxO signaling pathway	4	0.002	IL6, CCND1, PLK1, SOD2
**hsa04066**	HIF-1 signaling pathway	3	0.015	IL6, RPS6, GAPDH
**hsa04110**	Cell cycle	3	0.025	CCND1, PLK1, CDK1

Enrichment analysis was done using DAVID and set count >2 and $p < 0.05$ as the cut off for significant enrichment. List of hub genes enriched in different pathways are presented. Up-regulated genes are marked in red; and down-regulated ones in black.

Supplementary Table S10: Hub genes and VEGF pathway association

VEGF pathway module	hub genes
ERK	RPS3, EGFR, UBA52, CXCL8, IL6, VEGFA, PTGS2, PLK1, SQSTM1, SRC, APP, DNMT1, CCNB1
FAK	SRC, PTGS2, IL6, VEGFA, UBA52
JNK	IL6, VEGFA, PTGS2
NFKB	SRC, EGFR, UBE2C, VEGFA, IL6, UBA52, PTGS2, CXCL8, APP, SQSTM1 RPS3
P38 MAPK	APP, SQSTM1, IL6, VEGFA, SRC, PTGS2, EZH2, EGFR, CXCL8, CCNB1, UBA52
PI3K-AKT-MTOR	SRC, VEGFA, EZH2, IL6, POLR2H, APP, UBA52, EGFR, PTGS2, SQSTM1, IDH1, CCNB1, CXCL8
PLC-PKC	VEGFA, EGFR, SQSTM1, SRC, UBE2C, UBA52, IL6
RAC	IL6, SRC, VEGFA, PLK1, CXCL8, EGFR
RHOA	IL6, NCL, VEGFA, EZH2
STAT	IL6, VEGFA, SRC, PTGS2, EZH2, CXCL8, EGFR, DNMT

The interaction between hub genes and the genes in the VEGF pathway was analysed using the different network modules in the VEGF mediated angiogenesis signaling pathway (Abhinand et al. 2016, Sunitha et al. 2019). The modules and the interacted hub genes are presented.
